# Post-resuscitation Critical Illness Dysphagia: A Case Report

**DOI:** 10.7759/cureus.86640

**Published:** 2025-06-24

**Authors:** Ahmed M M Hassan, Shaili Shah, Nikki Bustamante, Jhiamluka Solano

**Affiliations:** 1 Internal Medicine, Northern Lincolnshire and Goole National Health Service (NHS) Trust, Scunthorpe, GBR; 2 Resident Doctor Committee, Royal College of Physicians, London, GBR; 3 Education Committee, Academy of Medical Educators, Cardiff, GBR; 4 Cardiology, Scunthorpe General Hospital, Scunthorpe, GBR

**Keywords:** critical illness dysphagia, intensive care unit, mechanical ventilation, multidisciplinary management, swallowing assessment

## Abstract

Critical illness dysphagia (CID) is a common complication affecting patients following prolonged intensive care unit (ICU) admissions, particularly those who undergo endotracheal intubation and mechanical ventilation. CID is associated with increased morbidity, including aspiration pneumonia, prolonged hospitalisation, and the need for enteral nutrition. The aetiology is multifactorial, involving oropharyngeal trauma, neuromuscular weakness, neurological insult, and disuse atrophy. We report the case of a male patient in his sixties admitted with fluid overload who subsequently experienced a pulseless electrical activity (PEA) cardiac arrest, requiring ICU admission, mechanical ventilation, and inotropic support. His ICU course was complicated by staphylococcal bacteraemia and multi-organ dysfunction. Following extubation, he developed severe dysphagia. Structural and neurological causes were excluded through laryngoscopy, MRI of the brain, and autoimmune screening. Serial swallowing assessments, including fibreoptic endoscopic evaluation of swallowing (FEES) and videofluoroscopic swallow study (VFSS), confirmed profound pharyngeal dysphagia with silent aspiration. He required prolonged nasogastric feeding. Multidisciplinary care involving gastroenterology, neurology, endocrinology, speech and language therapy (SALT), and dietetics guided a structured rehabilitation programme. Despite initial deterioration, the patient showed gradual improvement with ongoing therapy, eventually progressing to a safe oral intake on a modified International Dysphagia Diet Standardisation Initiative (IDDSI) level 2-3 diet. He was discharged home with outpatient SALT and nutrition support. This case highlights the multifaceted nature of CID and the crucial role of early identification, multidisciplinary coordination, and structured dysphagia rehabilitation in enhancing functional outcomes. Instrumental assessments such as FEES and VFSS are key to guiding clinical decisions. With appropriate intervention, patients with CID can achieve meaningful recovery of swallowing function and avoid long-term enteral feeding.

## Introduction

Critical illness dysphagia (CID) is a common and multifactorial complication that occurs in up to 18.3% of patients following prolonged ICU stays, particularly in those who have undergone endotracheal intubation and mechanical ventilation [1]. It is associated with poor clinical outcomes, including prolonged hospitalisation, increased risk of aspiration pneumonia, need for enteral feeding, and higher mortality [2]. The aetiology of CID is complex and typically multifactorial, involving both central and peripheral mechanisms. Proposed contributing factors include oropharyngeal and laryngeal trauma from prolonged intubation, ICU-acquired weakness affecting swallowing musculature, neurological impairment (including hypoxic encephalopathy and drug effects), disuse atrophy due to NBM (nil-by-mouth) status and ventilator support, gastroesophageal reflux and delayed gastric emptying, and pre-existing comorbidities and age-related physiological decline [3-5]. Diagnosis requires multidisciplinary assessment using tools such as fiberoptic endoscopic evaluation of swallowing (FEES) and videofluoroscopic swallow studies (VFSS). Early recognition and therapy are essential to prevent complications like aspiration pneumonia, malnutrition, and delayed recovery [1,6].

## Case presentation

A male patient in his 60s was admitted with clinical features of fluid overload, including reduced air entry over both lung bases, tense ascites, and bilateral lower limb and scrotal oedema. Laboratory investigations revealed anaemia without evidence of active bleeding. During admission, he experienced a pulseless electrical activity (PEA) cardiac arrest, with an estimated downtime of 10 minutes. He was successfully resuscitated and transferred to the intensive care unit (ICU) for post-arrest care. His ICU stay was complicated by multi-organ involvement. He required 11 days of mechanical ventilation, inotropic support, and drainage of significant ascitic fluid. Microbiological cultures of the ascitic fluid isolated *Staphylococcus *and *Enterococcus* species, resulting in staphylococcal bacteraemia. He received targeted intravenous antibiotics and antifungal therapy, leading to gradual clinical improvement and step-down to a general medical ward.

Following extubation, the patient developed severe dysphagia. Neurological and structural causes were systematically excluded: myasthenia gravis antibody testing was negative, brain MRI was unremarkable, and laryngoscopy showed no anatomical abnormalities. Swallowing function was serially assessed by the speech and language therapy (SALT) team. The initial fibreoptic endoscopic evaluation of swallowing (FEES) on 17 December 2024 revealed moderately severe pharyngeal dysphagia, with silent aspiration of International Dysphagia Diet Standardisation Initiative (IDDSI) level 2 liquids and level 4 food, a high secretion burden, and poor airway protection. Nasogastric (NG) feeding was continued.

A follow-up FEES on 23 December 2024 demonstrated deterioration, with severe dysphagia and silent aspiration across all consistencies, along with persistent pharyngeal residue. There was no indication to progress to oral intake, and NG feeding remained in place. Care was coordinated by a multidisciplinary team (MDT) involving gastroenterology, neurology, endocrinology, SALT, and dietetics. Percutaneous endoscopic gastrostomy (PEG) was considered; however, the patient declined the intervention. A videofluoroscopic swallow study (VFSS) on 6 January 2025 confirmed ongoing severe dysphagia involving both oral and pharyngeal phases, with no significant improvement despite intensive SALT input (see Video 1). Nutritional support continued via the NG route.

**Video 1:** Videofluoroscopic swallow study (VFSS)

A repeat FEES on 14 January 2025 demonstrated moderate dysphagia with improved airway protection. Limited oral trials with IDDSI level 2-3 consistencies were initiated under supervision by the speech and language therapists. Although some progress was evident, NG feeding remained the primary nutritional route at that stage. With ongoing therapy, the patient showed gradual improvement, eventually tolerating a supervised IDDSI level 2-3 diet. Nutritional requirements were met orally, allowing for the safe removal of the NG tube. The patient regained safe swallowing function and was able to meet his nutritional needs on a modified diet. He was discharged home with continued support from the outpatient SALT and community nutrition teams.

## Discussion

This case illustrates the complexity of diagnosing and managing critical illness dysphagia (CID). The pathophysiology of CID is multifactorial, with both central and peripheral mechanisms contributing to its development. Prolonged intubation and mechanical ventilation can result in trauma to the oropharyngeal and laryngeal structures, leading to dysfunction in swallowing [4]. Additionally, ICU-acquired weakness, which affects the musculature involved in swallowing, further exacerbates the condition [3]. Swallowing begins in the cortex and is coordinated by the medullary swallowing centre, which activates cranial nerves (V, VII, IX, X, XII) and spinal nerves (C1-C3) to control the swallowing muscles. Sensory input from the oropharynx, larynx, and oesophagus, transmitted via cranial nerves V, IX, and X, forms a feedback loop with the cortex. Critical illness dysphagia disrupts this pathway at multiple levels, central, peripheral, and sensory, impairing the swallowing reflex and contributing to dysfunction in critically ill patients [1]. 

Furthermore, critical illness neuropathy (CIN) and critical illness myopathy (CIM) are important contributors to swallowing dysfunction in ICU patients. These neuromuscular complications, often under-recognised, can lead to impaired coordination and strength of the oropharyngeal and laryngeal muscles, thereby increasing the risk of dysphagia, aspiration, and prolonged ventilator dependency [1-3]. The prevalence of post-extubation dysphagia has been linked to the duration of mechanical ventilation, sedation, and neuromuscular impairment, all of which are common in patients with CIN/CIM [4-6]. Specifically, Schefold et al. found an 18.3% incidence in an unselected ICU population and 10.3% at ICU discharge with clinically significant dysphagia, often associated with underlying neuromuscular disorders [5]. Furthermore, Macht et al. demonstrated that dysphagia in critically ill patients with neurologic deficits, including those stemming from CIN and CIM, was associated with increased hospital length of stay and morbidity [7]. These findings underscore the need for routine assessment of swallowing function following extubation, particularly in patients with identified risk factors for CIN/CIM.

Additionally, neurological complications, including hypoxic encephalopathy and the effects of sedative medications, can contribute to swallowing deficits [8]. Disuse atrophy due to NBM (nil-by-mouth) status and the use of mechanical ventilation are also significant contributors to dysphagia in the ICU setting. Furthermore, gastroesophageal reflux disease (GERD) and delayed gastric emptying are frequently observed in ICU patients and may exacerbate swallowing difficulties by increasing the risk of aspiration [9].

The patient's case underscores the importance of early and thorough evaluation using diagnostic tools such as FEES and VFSS to inform management decisions [6]. Prompt identification of swallowing dysfunction enables timely intervention, including dietary modifications and the use of enteral feeding when necessary. A multidisciplinary approach, incorporating gastroenterologists, speech and language therapists, and neurologists, is crucial for a comprehensive treatment approach, improving the chances of recovery and minimising the risk of complications such as aspiration pneumonia. The patient's outcome aligns with the growing body of evidence supporting the role of structured dysphagia therapy in improving swallowing function following critical illness [7]. With appropriate intervention, many patients with CID can regain functional swallowing ability and achieve nutritional goals orally, as demonstrated in this case.

## Conclusions

This case highlights the multifactorial nature of critical illness dysphagia (CID) and underscores the importance of a multidisciplinary approach to its diagnosis and management. The patient's recovery trajectory, marked by prolonged mechanical ventilation, post-extubation dysphagia, and gradual functional improvement through coordinated input from speech and language therapy, dietetics, gastroenterology, neurology, and endocrinology, illustrates the complexity of CID. Early identification using instrumental assessments such as FEES and VFSS was instrumental in guiding nutritional and therapeutic interventions. With structured, tailored rehabilitation, the patient achieved safe oral intake and was discharged without the need for long-term enteral feeding. This case highlights the importance of early, individualised, and multidisciplinary strategies to enhance outcomes and promote recovery in patients with CID.
